# Local Reasoning for Global Graph Properties

**DOI:** 10.1007/978-3-030-44914-8_12

**Published:** 2020-04-18

**Authors:** Siddharth Krishna, Alexander J. Summers, Thomas Wies

**Affiliations:** ETH Zurich, Zurich, Switzerland; 9 New York University, New York, NY USA; 10 ETH Zürich, Zurich, Switzerland

## Abstract

Separation logics are widely used for verifying programs that manipulate complex heap-based data structures. These logics build on so-called *separation algebras*, which allow expressing properties of heap regions such that modifications to a region do not invalidate properties stated about the remainder of the heap. This concept is key to enabling modular reasoning and also extends to concurrency. While heaps are naturally related to mathematical graphs, many ubiquitous graph properties are non-local in character, such as reachability between nodes, path lengths, acyclicity and other structural invariants, as well as data invariants which combine with these notions. Reasoning modularly about such graph properties remains notoriously difficult, since a local modification can have side-effects on a global property that cannot be easily confined to a small region.

In this paper, we address the question: What separation algebra can be used to avoid proof arguments reverting back to tedious global reasoning in such cases? To this end, we consider a general class of global graph properties expressed as fixpoints of algebraic equations over graphs. We present mathematical foundations for reasoning about this class of properties, imposing minimal requirements on the underlying theory that allow us to define a suitable separation algebra. Building on this theory, we develop a general proof technique for modular reasoning about global graph properties expressed over program heaps, in a way which can be directly integrated with existing separation logics. To demonstrate our approach, we present local proofs for two challenging examples: a priority inheritance protocol and the non-blocking concurrent Harris list.

### 1 Introduction

Separation logic (SL) [31,37] provides the basis of many successful verification tools that can verify programs manipulating complex data structures [1,4,17,29]. This success is due to the logic's support for reasoning modularly about modifications to heap-based data. For simple inductive data structures such as lists and trees, much of this reasoning can be automated [2,11,20,33]. However, these techniques often fail when data structures are less regular (e.g. multiple overlaid data structures) or provide multiple traversal patterns (e.g. threaded trees). Such idioms are prevalent in real-world implementations such as the fine-grained concurrent data structures found in operating systems and databases. Solutions to these problems have been proposed [14] but remain difficult to automate. For proofs of general graph algorithms, the situation is even more dire. Despite substantial improvements in the verification methodology for such algorithms [35,38], significant parts of the proof argument still typically need to be carried out using non-local reasoning [7,8,13,25]. This paper presents a general technique for local reasoning

```
1 method acquire(p: Node, r: Node) {
2   if (r.next == null) {
3     r.next := p; update(p, -1, r.curr_prio)
4   } else {
5     p.next := r; update(r, -1, p.curr_prio)
6   }
7 }
8 method update(n: Node, from: Int, to: Int) {
9   n.prios := n.prios \ {from}
10  if (to >= 0) n.prios := n.prios ∪ {to}
11  from := n.curr_prio
12  n.curr_prio := max(n.prios ∪ {n.def_prio})
13  to := n.curr_prio;
14  if (from != to && n.next != null) {
15    update(n.next, from, to)
16  }
17 }
```

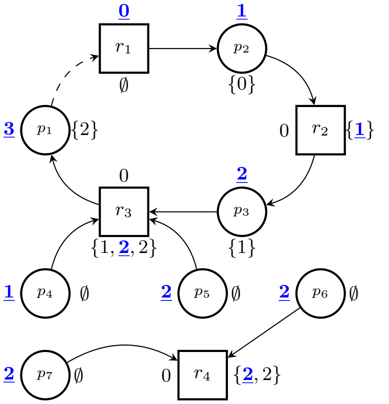

Fig. 1: Pseudocode of the PIP and a state of the protocol data structure. Round nodes represent processes and rectangular nodes resources. Nodes are marked with their default priorities `def_prio` as well as the aggregate priority multiset `prios`. A node's current priority `curr_prio` is underlined and marked in bold blue.

about global graph properties that can be used within off-the-shelf separation logics. We demonstrate our technique using two challenging examples for which no fully local proof existed before, respectively, whose proof required a tailor-made logic.

As a motivating example, we consider an idealized priority inheritance protocol (PIP), a technique used in process scheduling [39]. The purpose of the protocol is to avoid *priority inversion*, i.e. a situation where a low-priority process causes a high-priority process to be blocked. The protocol maintains a bipartite graph with nodes representing processes and resources. An example graph is shown in Fig. 1. An edge from a process $p$ to a resource $r$ indicates that $p$ is waiting for $r$ to be available whereas an edge in the other direction means that $r$ is currently held by $p$. Every node has an associated *default* priority and *current*; these are natural numbers. The current priority is used for scheduling processes. When a process attempts to acquire a resource currently held by another process, the graph is updated to avoid priority inversion. For example, when process $p_1$ with current priority 3 attempts to acquire the resource $r_1$ held by process $p_2$ of priority 1, $p_1$'s higher priority is propagated to $p_2$ and, transitively, to any other process that $p_2$ is waiting for ($p_3$ in this case). As a result, all nodes on the created cycle[3] will get current priority 3. The protocol maintains the following *invariant*: the current priority of each node is the maximum of its default priority and the current priorities of all its predecessors. Priority propagation is implemented by the method `update` shown in Fig 1. The implementation represents graph edges by `next` pointers and handles both adding an edge (`acquire`) and removing one (`release` - code omitted). To recalculate the current priority of a node (line 12), each node maintains its default priority `def_prio` and a multiset `prios` which contains the priorities of all its immediate predecessors.

Verifying that the PIP maintains its invariant using established separation logic (SL) techniques is challenging. In general, SL assertions describe resources and express the fact that the program has permission to access and manipulate these resources. In what

[3] The cycle can be used to detect/handle a deadlock; this is not the concern of this data structure.

follows, we stick to the standard model of SL where resources are memory regions represented as partial heaps. We sometimes view partial heaps more abstractly as partial graphs (hereafter, simply graphs). Assertions describing larger regions are built from smaller ones using *separating conjunction*, $\phi_1 * \phi_2$. Semantically, the $*$ operator is tied to a notion of resource composition defined by an underlying *separation algebra* [5,6]. In the standard model, composition enforces that $\phi_1$ and $\phi_2$ must describe disjoint regions. The logic and algebra are set up so that changes to the region $\phi_1$ do not affect $\phi_2$ (and vice versa). That is, if $\phi_1 * \phi_2$ holds before the modification and $\phi_1$ is changed to $\phi_1'$, then $\phi_1' * \phi_2$ holds afterwards. This so-called *frame rule* enables modular reasoning about modifications to the heap and extends well to the concurrent setting when threads operate on disjoint portions of memory [3,9,10,36]. However, the mere fact that $\phi_2$ is preserved by modifications to $\phi_1$ does not guarantee that if a global property such as the PIP invariant holds for $\phi_1 * \phi_2$, it also still holds for $\phi_1' * \phi_2$.

For example, consider the PIP scenario depicted in Fig. 1. If $\phi_1$ describes the subgraph containing only node $p_1$, $\phi_2$ the remainder of the graph, and $\phi_1'$ the graph obtained from $\phi_1$ by adding the edge from $p_1$ to $r_1$, then the PIP invariant will no longer hold for the new composed graph described by $\phi_1' * \phi_2$. On the other hand, if $\phi_1$ captures $p_1$ and the nodes reachable from $r_1$ (i.e., the set of nodes modified by `update`), $\phi_2$ the remainder of the graph, and we reestablish the PIP invariant locally in $\phi_1$ obtaining $\phi_1'$ (i.e., run `update` to completion), then $\phi_1' * \phi_2$ will also globally satisfy the PIP invariant. The separating conjunction $*$ is not sufficient to differentiate these two cases; both describe valid partitions of a possible program heap. As a consequence, prior techniques have to revert back to non-local reasoning to prove that the invariant is maintained.

A first helpful idea towards a solution to this problem is that of *iterated separating conjunction* [30,44], which describes a graph $G$ consisting of a set of nodes $X$ by a formula $\Psi = \mathop{\Large\ast}_{x \in X} \mathsf{N}(x)$ where $\mathsf{N}(x)$ is some predicate that holds locally for every node $x \in X$. Using such node-local conditions one can naturally express non-inductive properties of graphs (e.g. *"G has no outgoing edges"* or *"G is bipartite"*). The advantages of this style of specification are two-fold. First, one can arbitrarily decompose and recompose $\Psi$ by splitting $X$ into disjoint subsets. For example, if $X$ is partitioned into $X_1$ and $X_2$, then $\Psi$ is equivalent to $\mathop{\Large\ast}_{x \in X_1} \mathsf{N}(x) * \mathop{\Large\ast}_{x \in X_2} \mathsf{N}(x)$. Moreover, it is very easy to prove that $\Psi$ is preserved under modifications of subgraphs. For instance, if a program modifies the subgraph induced by $X_1$ such that $\mathop{\Large\ast}_{x \in X_1} \mathsf{N}(x)$ is preserved locally, then the frame rule guarantees that $\Psi$ will be preserved in the new larger graph. Iterated separating conjunction thus yields a simple proof technique for local reasoning about graph properties that can be described in terms of node-local conditions. However, this idea alone does not actually solve our problem because general global graph properties such as *"G is a direct acyclic graph"*, *"G is an overlay of multiple trees"*, or *"G satisfies the PIP invariant"* cannot be directly described via node-local conditions.

*Solution.* The key ingredient of our approach is the concept of a *flow* of a graph: a function *fl* from the nodes of the graph to *flow values*. For the PIP, the flow maps each node to the multiset of its incoming priorities. In general, a flow is a fixpoint of a set of algebraic equations induced by the graph. These equations are defined over a *flow domain*, which determines how flow values are propagated along the edges of the graph and how they are aggregated at each node. In the PIP example, an edge between nodes $(n, n')$ propagates the multiset containing $\max(\mathit{fl}(n), n.\texttt{def\_prio})$ from $n$ to $n'$. The multisets arriving at $n'$ are aggregated with multiset union to obtain $\mathit{fl}(n')$. Flows enable capturing global graph properties in terms of node-local conditions. For example, the PIP invariant can be expressed by the following node-local condition: $n.\texttt{curr\_prio} = \max(\mathit{fl}(n), n.\texttt{def\_prio})$. To enable compositional reasoning about such properties we need an appropriate separation algebra allowing us to prove locally that modifications to a subgraph do not affect the flow of the remainder of the graph.

To this end, we make the useful observation that a separation algebra induces a notion of an *interface of a resource*: we say that two resources $a$ and $a'$ are equivalent if they compose with the same resources. The interface of a resource $a$ could then be defined as $a$'s equivalence class, but more-succinct and simpler representations may be possible. In the standard model of SL where resources are graphs and composition is disjoint graph union, the interface of a graph $G$ is the set of all graphs $G'$ that have the same domain as $G$; in this model, a graph's domain could be defined to be its interface.

The interfaces of resources described by assertions capture the information that is implicitly communicated when these assertions are conjoined by separating conjunction. As we discussed earlier, in the standard model of SL, this information is too weak to enable local reasoning about global properties of the composed graphs because some additional information about the subgraphs' structure other than which nodes they contain must be communicated. For instance, if the goal is to verify the PIP invariant, the interfaces must capture information about the multisets of priorities propagated between the subgraphs. We define a separation algebra achieving exactly this: the induced *flow interface* of a graph $G$ in this separation algebra captures how values of the flow domain must enter and leave $G$ such that, when composed with a compatible graph $G'$, the imposed local conditions on the flow of each node are satisfied in the composite graph.

This is the key to enabling SL-style framing for global graph properties. Using iterated separating conjunctions over the new separation algebra, we obtain a compositional proof technique that yields succinct proofs of programs such as the PIP, whose proofs with existing techniques would involve non-trivial global reasoning steps.

*Contributions.* In §2, we present mathematical foundations for flow domains, imposing the minimal requirements on the underlying algebra that allow us to capture a broad range of data structure invariants and graph properties and reason locally about them in a suitable separation algebra. Building on this theory we develop a general proof technique for modular reasoning about global graph properties that can be integrated with existing separation logics (§3). We further identify general mathematical conditions that can be used when desired to guarantee unique flows, and provide local proof arguments to check the preservation of these conditions (§4). We demonstrate the versatility of our approach by presenting local proofs for two challenging examples: the PIP and the concurrent non-blocking list due to Harris [12].

*Flows Redesigned.* Our work is inspired by the recent flow framework explored by some of the authors [22], but was redesigned from the ground up. We revisit the core algebra behind flow reasoning, and derive a different algebraic foundation by analysing the minimal requirements for general local reasoning; we call our newly-designed reasoning framework the *foundational flow framework*. Our new framework makes several significant improvements over [22] and eliminates its most stark limitations. We provide a detailed technical comparison with [22] and discuss other related work in §5.

### 2 The Foundational Flow Framework

In this section, we introduce the foundational flow framework, explaining the motivation for its design with respect to local reasoning principles. We aim for a general technique for modularly proving the preservation of recursively-defined invariants over (partial) graphs, with well-defined decomposition and composition operations.

#### 2.1 Preliminaries and Notation

The term $(b\ ?\ t_1 : t_2)$ denotes $t_1$ if condition $b$ holds and $t_2$ otherwise. We write $f \colon A \to B$ for a function from $A$ to $B$, and $f \colon A \rightharpoonup B$ for a partial function from $A$ to $B$. For a partial function $f$, we write $f(x) = \bot$ if $f$ is undefined at $x$. We use lambda notation $(\lambda x.\ E)$ to denote a function that maps $x$ to the expression $E$ (typically containing $x$). If $f$ is a function from $A$ to $B$, we write $f[x \rightarrowtail y]$ to denote the function from $A \cup \{x\}$ defined by $f[x \rightarrowtail y](z) := (z = x\ ?\ y : f(z))$. We use $\{x_1 \rightarrowtail y_1, \ldots, x_n \rightarrowtail y_n\}$ for pairwise different $x_i$ to denote the function $\epsilon[x_1 \rightarrowtail y_1] \cdots [x_n \rightarrowtail y_n]$, where $\epsilon$ is the function on an empty domain. Given functions $f_1 \colon A_1 \to B$ and $f_2 \colon A_2 \to B$ we write $f_1 \uplus f_2$ for the function $f \colon A_1 \uplus A_2 \to B$ that maps $x \in A_1$ to $f_1(x)$ and $x \in A_2$ to $f_2(x)$ (if $A_1$ and $A_2$ are not disjoint sets, $f_1 \uplus f_2$ is undefined).

We write $\delta_{n=n'} \colon M \to M$ for the function defined by $\delta_{n=n'}(m) := m$ if $n = n'$ else 0. We also write $\lambda_0 := (\lambda m.\ 0)$ for the identically zero function, $\lambda_{\mathsf{id}} := (\lambda m.\ m)$ for the identity function, and use $e \equiv e'$ to denote function equality. For $e \colon M \to M$ and $m \in M$ we write $m \triangleright e$ to denote the function application $e(m)$. We write $e \circ e'$ to denote function composition, i.e. $(e \circ e')(m) = e(e'(m))$ for $m \in M$, and use superscript notation $e^p$ to denote the function composition of $e$ with itself $p$ times.

For multisets $S$, we use standard set notation when clear from the context. We write $S(x)$ to denote the number of occurrences of $x$ in $S$. We write $\{x_1 \rightarrowtail i_1, \ldots, x_n \rightarrowtail i_n\}$ for the multiset containing $i_1$ occurrences of $x_1$, $i_2$ occurrences of $x_2$, etc.

A *partial monoid* is a set $M$, along with a partial binary operation $+ \colon M \times M \rightharpoonup M$, and a special zero element $0 \in M$, such that (1) $+$ is associative, i.e., $(m_1 + m_2) + m_3 = m_1 + (m_2 + m_3)$; and (2) 0 is an identity, i.e., $m + 0 = 0 + m = m$. Here, $=$ means either both sides are defined and equal, or both are undefined. We identify a partial monoid with its support set $M$. If $+$ is a total function, then we call $M$ a monoid. Let $m_1, m_2, m_3 \in M$ be arbitrary elements of the (partial) monoid in the following. We call a (partial) monoid $M$ *commutative* if $+$ is commutative, i.e., $m_1 + m_2 = m_2 + m_1$. Similarly, a commutative monoid $M$ is *cancellative* if $+$ is cancellative, i.e., if $m_1 + m_2 = m_1 + m_3$ is defined, then $m_2 = m_3$.

A *separation algebra* [5] is a cancellative, partial, commutative monoid.

#### 2.2 Flows

Recursive properties of graphs naturally depend on non-local information; e.g. we cannot express that a graph is acyclic directly as a conjunction of per-node invariants. Our foundational flow framework defines *flow values* at each node that capture non-local graph properties, and enables local specification and reasoning about such properties. Flow values are drawn from a *flow domain*, an algebraic structure which also specifies the operations used to define a flow via recursive computations over the graph. Our entire theory is parametric with the choice of a flow domain, whose components will be explained and motivated in the rest of this section.

**Definition 1 (Flow Domain).** *A* flow domain $(M, +, 0, E)$ *consists of a commutative cancellative (total) monoid* $(M, +, 0)$ *and a set of* edge functions $E \subseteq M \to M$.

*Example 1.* The *path-counting* flow domain is $(\mathbb{N}, +, 0, \{\lambda_{\mathsf{id}}, \lambda_0\})$, consisting of the monoid of natural numbers under addition and the set of edge functions containing only the identity function and the zero function. This can be used to define a flow where the values at each node represent the number of paths to this node from a distinguished node $n$. Path-counting provides enough information to express locally per node that e.g. (a) all nodes are reachable from $n$ (all path counts are non-zero), or (b) that the graph forms a tree rooted at $n$ (all path counts are exactly 1).

*Example 2.* We use $(\mathbb{N}^{\mathbb{N}}, \cup, \emptyset, \{\lambda_0\} \cup \{(\lambda m.\ \{\max(m \cup \{p\})\}) \mid p \in \mathbb{N}\})$ as flow domain for the PIP example (Figure 1). This consists of the monoid of multisets of natural numbers under multiset union and two kinds of edge functions: $\lambda_0$ and functions mapping a multiset $m$ to the singleton multiset containing the maximum value between $m$ and a fixed value $p$ (used to represent a node's default priority). This can define a flow which locally captures the appropriate current node priorities as the graph is modified.

Further definitions in this section assume a fixed flow domain $(M, +, 0, E)$ and a (potentially infinite) set of nodes $\mathfrak{N}$. For this section, we abstract heaps using directed partial graphs; integration of our graph reasoning with direct proofs over program heaps is explained in §3.

**Definition 2 (Graph).** *A* (partial) graph $G = (N, e)$ *consists of a finite set of nodes* $N \subseteq \mathfrak{N}$ *and a mapping from pairs of nodes to edge functions* $e \colon N \times \mathfrak{N} \to E$.

*Flow Values and Flows.* Flow values (taken from $M$; the first element of a flow domain) are used to capture sufficient information to express desired non-local properties of a graph. In Example 1, flow values are non-negative integers; for the PIP (Example 2) we instead use *multisets* of integers, representing relevant *non-local* information: the priorities of nodes currently referencing a given node in the graph. Given such flow values, a node's correct priority can be defined locally per node in the graph. This definition requires only the *maximum* value of these multisets, but as we will see shortly these multisets enable local *recomputation* of a correct priority when the graph is changed.

For a graph $G = (N, e)$ we express properties of $G$ in terms of node-local conditions that may depend on the nodes' *flow*. A flow is a function $\mathit{fl} \colon N \to M$ assigning every node a flow value and must be some fixpoint of the following *flow equation*:

$$\forall n \in N.\ \mathit{fl}(n) = \mathit{in}(n) + \sum_{n' \in N} \mathit{fl}(n') \triangleright e(n', n) \qquad \text{(FlowEqn)}$$

Intuitively, one can think of the flow as being obtained by a fold computation over the graph:[4] the *inflow* $in\colon N \to M$ defines an initial flow at each node. This initial flow is then updated recursively for each node $n$: the current flow value at its predecessor nodes $n'$ is transferred to $n$ via *edge functions* $e(n', n)\colon M \to M$. These flow values are aggregated using the *summation operation* $+$ of the flow domain to obtain an updated flow of $n$; a flow for the graph is some fixpoint satisfying this equation at all nodes. [5]

**Definition 3 (Flow Graph).** *A* flow graph $H = (N, e, \mathit{fl})$ *is a graph* $(N, e)$ *and function* $\mathit{fl}\colon N \to M$ *such that there exists an* inflow $in\colon N \to M$ *satisfying* $\mathsf{FlowEqn}(in, e, \mathit{fl})$.

We let $\mathsf{dom}(H) = N$, and sometimes identify $H$ and $\mathsf{dom}(H)$ to ease notational burden. For $n \in H$ we write $H_n$ for the singleton flow subgraph of $H$ induced by $n$.

*Edge Functions.* In any flow graph, the flow value assigned to a node $n$ by a flow is propagated to its neighbours $n'$ (and transitively) according to the edge function $e(n, n')$ labelling the edge $(n, n')$. The edge function maps the flow value at the *source node* $n$ to one propagated on *this edge* to the *target node* $n'$. Note that we require such a labelling for *all* pairs consisting of a source node $n$ inside the graph and a target node $n' \in \mathfrak{N}$ (i.e., possibly outside the graph). The 0 flow value (the third element of our flow domains) is used to represent no flow; the corresponding (constant) zero *function* $\lambda_0 = (\lambda m.\ 0)$ is used as edge function to model the *absence* of an edge in the graph. A set of edge functions $E$ from which this labelling is chosen can, other than the requirement $\lambda_0 \in E$, be chosen as desired. As we will see in §4.4, restrictions to particular sets of edge functions $E$ can be exploited to further strengthen our overall technique. Edge functions can depend on the local state of the source node (as in the following example); dependencies from elsewhere in the graph must be represented by the node's flow.

*Example 3.* Consider the graph in Figure 1 and the flow domain as in Example 2. We choose the edge functions to be $\lambda_0$ where no edge exists in the PIP structure, and otherwise $(\lambda m.\ \{\max(m \cup \{d\})\})$ where $d$ is the default priority of the source of the edge. For example, in Figure 1, $e(r_3, p_2) = \lambda_0$ and $e(r_3, p_1) = (\lambda m.\ \{\max(m \cup \{0\})\})$. Since the flow value at $r_3$ is $\{1, 2, 2\}$, the edge $(r_3, p_1)$ propagates the value $\{2\}$ to $p_1$, correctly representing the current priority of $r_3$.

*Flow Aggregation and Inflows.* The flow value at a node is defined by those propagated to it from each node in a graph via edge functions, along with an additional *inflow* value explained here. Since multiple non-zero flow values can be propagated to a node, we require an aggregation of these values via a binary $+$ operator on flow values : the second element of our flow domains. The edges from which the aggregated values originate are unordered. Thus, we require $+$ to be commutative and associative, making this aggregation order-independent. The 0 flow value must act as a unit for $+$. For example, in the path-counting flow domain $+$ means addition on natural numbers, while for the multisets employed for the PIP it means multiset union.

[4] We note that flows are not generally defined in this manner as we consider any fixpoint of the flow equation to be a flow. Nonetheless, the analogy helps to build an initial intuition.

[5] We discuss questions regarding the existence and uniqueness of such fixpoints in §4.

Each node in a flow graph has an *inflow*, modelling contributions to its flow value which do *not* come from inside the graph. Inflows play two important roles: first, since our graphs are partial, they model contributions from nodes *outside of the graph*. Second, inflow can be artificially added as a means of specialising the computation of flow values to characterise specific graph properties. For example, in the path-counting domain, we give an inflow of 1 to the node from which we are counting paths, and 0 to all others.

*Example 4.* Let the edges in the graph in Figure 1 be labelled as described in Example 3. If the inflow function $in$ assigns the empty multiset to every node $n$ and we let $\mathit{fl}(n)$ be the multiset labelling every node in the figure, then $\mathsf{FlowEqn}(in, e, \mathit{fl})$ holds.

The flow equation $(\mathsf{FlowEqn})$ defines the flow of a node $n$ to be the aggregation of flow values coming from other nodes $n'$ inside the graph (as given by the respective edge function $e(n', n)$) as well as the inflow $in(n)$. Preserving solutions to this equation across updates to the graph structure is a fundamental goal of our technique. The following lemma (which relies on the fact that $+$ is required to be cancellative) states that any correct flow values uniquely determine appropriate inflow values:

**Lemma 1.** *Given a flow graph* $(N, e, \mathit{fl})$*, there exists a unique inflow* $in$ *such that* $\mathsf{FlowEqn}(in, e, \mathit{fl})$.

We now turn to how solutions of the flow equation can be preserved or appropriately updated under *changes* to the underlying graph.

*Graph Updates and Cancellativity.* Given a flow graph with known flow and inflow values, suppose we *remove* an edge from $n_1$ to $n_2$ (replacing the edge function with $\lambda_0$). For the same inflow, such an update will potentially affect the flow at $n_2$ and nodes to which $n_2$ (transitively) propagates flow. Starting from the simple case that $n_2$ has no outgoing edges, we need to recompute a suitable flow at $n_2$. Knowing the old flow value (say, $m$) and the contribution $m' = \mathit{fl}(n_1) \triangleright e(n_1, n_2)$ *previously* provided along the removed edge, we know that the correct new flow value is some $m''$ such that $m' + m'' = m$. This constraint has a unique solution (and thus, we can unambiguously recompute a new flow value) exactly when the aggregation $+$ is *cancellative*; we therefore make cancellativity a *requirement* on the $+$ of any flow domain.

Cancellativity intuitively enforces that the flow domain carries enough information to enable adaptation to local updates (in particular, removal of edges[6]). Returning to the PIP example, cancellativity requires us to carry multisets as flow values rather than only the maximum priority value: $+$ cannot be the maximum operation, as this would not be cancellative. The resulting multisets (like the `prio` fields in the actual code) provide the information necessary to recompute corrected priority values locally.

For example, in the PIP graph shown in Figure 1, removing the edge from $p_6$ to $r_4$would not affect the current priority of $r_4$ whereas if $p_7$ had current priority 1 instead of 2, then the current priority of $r_4$ would have to decrease. In either case, recomputing the flow value for $r_4$ is simply a matter of subtraction (removing $\{2\}$ from the multiset at $r_4$); cancellativity guarantees that our flow domains will always provide the information

[6] As we will show in §2.3, an analogous problem for composition of flow graphs is also directly solved by this choice to force aggregation to be cancellative.

needed for this recomputation. Without this property, the recomputation of a flow value for the target node $n_2$ would, in general, entail recomputing the incoming flow values from all remaining edges from scratch. Cancellativity is also crucial for Lemma 1 above, forcing uniqueness of inflows, given known flow values in a flow graph. This allows us to define natural but powerful notions of flow graph decomposition and recomposition.

#### 2.3 Flow Graph Composition and Abstraction

Building towards the core of our reasoning technique, we now turn to the question of decomposition and recomposition of flow graphs. Two flow graphs with disjoint domains always compose to a graph, but this will be a *flow graph* only if their flows are chosen consistently to admit a solution to the resulting flow equation (i.e. the flow graph composition operator $\odot$ defined below is *partial*).

**Definition 4 (Flow Graph Algebra).** *The* flow graph algebra $(\mathsf{FG}, \odot, H_\emptyset)$ *for the flow domain* $(M, +, 0, E)$ *is defined by*

$$\mathsf{FG} := \{(N, e, \mathit{fl}) \mid (N, e, \mathit{fl}) \text{ is a flow graph}\}, \qquad H_\emptyset := (\emptyset, e_\emptyset, \mathit{fl}_\emptyset),$$
$$(N_1, e_1, \mathit{fl}_1) \odot (N_2, e_2, \mathit{fl}_2) := \begin{cases} (N_1 \uplus N_2, e_1 \uplus e_2, \mathit{fl}_1 \uplus \mathit{fl}_2) & \text{if in } \mathsf{FG} \\ \bot & \text{otherwise,} \end{cases}$$

*where* $e_\emptyset$ *and* $\mathit{fl}_\emptyset$ *are the edge functions and flow on the empty set of nodes* $N = \emptyset$.

Intuitively, two flow graphs compose to a flow graph if their contributions to each others' flow (along edges from one to the other) are reflected in the corresponding inflow of the other graph. For example, consider the subgraph from Figure 1 consisting of the single node $p_7$ (with 0 inflow). This will compose with the remainder of the graph depicted only if this remainder subgraph has an inflow which, at node $r_4$, includes at least the multiset $\{2\}$, reflecting the propagated value from $p_7$.

We use this intuition to extract an *abstraction* of flow graphs which we call *flow interfaces*. Given a flow (sub)graph, its flow interface consists of the node-wise inflow and *outflow* (the flow contributions its nodes make to all nodes outside of the graph, defined below). It is thus an abstraction that hides the flow values and edges that are wholly *inside* the flow graph. Flow graphs that have the same flow interface "look the same" to the external graph, as the same values are propagated inwards and outwards.

**Definition 5 (Flow Interface).** *For a given flow domain* $M$*, a* flow interface *is a pair* $I = (\mathit{in}, \mathit{out})$ *where* $\mathit{in}\colon N \to M$ *and* $\mathit{out}\colon \mathfrak{N} \setminus N \to M$ *for some* $N \subseteq \mathfrak{N}$.

We write $I.\mathit{in}, I.\mathit{out}$ for the two components of the interface $I = (\mathit{in}, \mathit{out})$. We will again sometimes identify $I$ and $\mathsf{dom}(I.\mathit{in})$ to ease notational burden.

Given a flow graph $H \in \mathsf{FG}$, we can compute its interface as follows. Recall that Lemma 1 implies that any flow graph has a unique inflow. Thus, we can define an inflow function that maps each flow graph $H = (N, e, \mathit{fl})$ to the unique inflow $\mathsf{inf}(H)\colon H \to M$ such that $\mathsf{FlowEqn}(\mathsf{inf}(H), e, \mathit{fl})$. Dually, we define the *outflow* of $H$ as the function $\mathsf{outf}(H)\colon \mathfrak{N} \setminus N \to M$ defined by $\mathsf{outf}(H)(n) := \sum_{n' \in N} \mathit{fl}(n') \triangleright e(n', n)$. The *flow interface of* $H$, written $\mathsf{int}(H)$, is the pair $(\mathsf{inf}(H), \mathsf{outf}(H))$ consisting of its inflow and its outflow. Returning to the previous example, if $H$ is the singleton subgraph consisting of node $p_7$ from Figure 1 with flow and edges as depicted, then $\mathsf{int}(H) = (\lambda n.\, \emptyset, \lambda n.\, (n{=}r_4 \; ? \; \{2\} : \emptyset))$.

This abstraction, while simple, turns out to be powerful enough to build a separation algebra over our flow graphs, allowing them to be decomposed, locally modified and recomposed in ways yielding all the local reasoning benefits of separation logics. In particular, for graph operations within a subgraph with a certain interface, we need to prove: (a) that the modified subgraph is still a flow graph (by checking that the flow equation still has a solution locally in the subgraph) and (b) that it satisfies the same interface (in other words, the effect of the modification on the flow is contained within the subgraph); the meta-level results for our technique then justify that we can recompose the modified subgraph with any graph that the original could be composed with.

We define the corresponding *flow interface algebra* as follows:

**Definition 6 (Flow Interface Algebra).** *For a given flow domain $M$, the* flow interface algebra *over $M$ is defined to be $(\mathsf{FI}, \oplus, I_\emptyset)$, where:*

$$\mathsf{FI} := \{I \mid I \text{ is a flow interface}\}, \qquad I_\emptyset \;:=\; \mathsf{int}(H_\emptyset),$$

$$I_1 \oplus I_2 := \begin{cases} I & I_1 \cap I_2 = \emptyset \\ & \quad \wedge \forall i \neq j \in \{1,2\}, n \in I_i.\; I_i.in(n) = I.in(n) + I_j.out(n) \\ & \quad \wedge \forall n \notin I.\; I.out(n) = I_1.out(n) + I_2.out(n) \\ \bot & \text{otherwise.} \end{cases}$$

Flow interface composition is well-defined because of cancellativity of the underlying flow domain (it is also, exactly as flow graph composition, partial). We next show the key result for this abstraction: the ability for two flow graphs to compose depends only on their interfaces; flow interfaces implicitly define a congruence relation on flow graphs.

**Lemma 2.** $\mathsf{int}(H_1) = I_1 \wedge \mathsf{int}(H_2) = I_2 \Rightarrow \mathsf{int}(H_1 \odot H_2) = I_1 \oplus I_2$.

Crucially, the following result shows that we can use our flow interfaces as an abstraction directly compatible with existing separation logics.

**Theorem 1.** *The flow interface algebra $(\mathsf{FI}, \oplus, I_\emptyset)$ is a separation algebra.*

This result forms the core of our reasoning technique; it enables us to make modifications within a chosen subgraph and, by proving preservation of its interface, know that the result composes with any context exactly as the original did. Flow interfaces capture precisely the information relevant about a flow graph, with respect to composition with other flow graphs. In Appendix B of the accompanying technical report (hereafter, TR) [23] we provide additional examples of flow domains that demonstrate the range of data structures and graph properties that can be expressed using flows, including a notion of *universal flow* that in a sense provides a completeness result for the expressivity of the framework. We now turn to constructing proofs atop these new reasoning principles.

## 3 Proof Technique

This section shows how to integrate flow reasoning into a standard separation logic, using the priority inheritance protocol (PIP) algorithm to illustrate our proof techniques.

Since flow graphs and flow interfaces form separation algebras, it is possible in principle to define a separation logic (SL) using these notions as a custom *semantic model* (indeed, this is the proof approach taken in [22]). By contrast, we integrate flow interfaces with a *standard* separation logic without modifying its semantics. This has the important technical advantage that our proof technique can be naturally integrated with existing separation logics and verification tools supporting SL-style reasoning. We consider a standard *sequential* SL in this section, but our technique can also be directly integrated with a concurrent SL such as RGSep (as we show in §4.5) or frameworks such as Iris [18] supporting (ghost) resources ranging over user-defined separation algebras.

### 3.1 Encoding Flow-based Proofs in SL

Proofs using our flow framework can employ a combination of specifications enforced at the node level and in terms of the flow graphs and interfaces corresponding to larger heap regions such as entire data structures (henceforth, *composite graphs* and *composite interfaces*). At the node level, we write invariants that every node is intended to satisfy, typically relating the node's flow value to its local state (fields). For example, in the PIP, we use node-local invariants to express that a node's current priority is the maximum of the node's default priority and those in its current flow value. We typically express such specifications in terms of *singleton (flow) graphs*, and their *singleton interfaces*.

Specification in terms of *composite* interfaces has several important purposes. One is to define custom inflows: e.g. in the path-counting flow domain, specifying that the inflow of a composite interface is 1 at some designated node $r$ and 0 elsewhere enforces in any underlying flow graph that each node $n$'s flow value will be the number of paths from $r$ to $n$.[7] Composite interfaces can also be used to express that, in two states of execution, a portion of the heap "looks the same" with respect to composition (it has the same interface, and so can be composed with the same flow graphs), or to capture by *how much* there is an observable difference in inflow or outflow; we employ this idea in the PIP proof below.

We now define an assertion syntax convenient for capturing both node-level and composite-level constraints, defined within an SL-style proof system. We assume an *intuitionistic*, *garbage-collected* SL [6] with standard syntax and semantics:[8] see Appendix A of the TR [23] for more details.

*Node Predicates.* The basic building block of our flow-based specifications is a node predicate $\mathsf{N}(x, H)$, representing ownership of the fields of a single node $x$, as well as

[7] Note that the analogous property cannot be captured at the node level; when considering singleton interfaces per node in a tree rooted at $r$, *every* singleton interface has an inflow of 1.

[8] As $P * \phi \equiv P \wedge \phi$ for pure formulas $P$ in garbage-collected SLs, we use $*$ instead of $\wedge$ throughout this paper.

capturing its corresponding singleton flow graph $H$:

$$\mathsf{N}(x, H) := \exists fs, fl.\ x \mapsto fs * H = (\{x\},\ (\lambda y.\ \mathsf{edge}(x, fs, y)),\ fl) * \gamma(x, fs, fl(x))$$

$\mathsf{N}$ is implicitly parameterised by $fs$, edge and $\gamma$; these are explained next and are typically fixed across any given flow-based proof. The $\mathsf{N}$ predicate expresses that we have a heap cell at location $x$ containing fields $fs$ (a list of field-name/value mappings).[9] It also says that $H$ is a singleton flow graph with domain $\{x\}$ with some flow $fl$, whose edge functions are defined by a user-defined abstraction function $\mathsf{edge}(x, fs, y)$; this function allows us to define edges in terms of $x$'s field values. Finally, the node, its fields, and its flow in this flow graph satisfy the custom predicate $\gamma$, used to encode node-local properties such as constraints in terms of the flow values of nodes.

*Graph Predicates.* The analogous predicate for composite graphs is Gr. It carries ownership to the nodes making up a potentially unbounded graph, using iterated separating conjunction over a *set* of nodes $X$ as mentioned in §1:

$$\mathsf{Gr}(X, H) := \exists \mathcal{H}.\ \mathop{\huge *}_{x \in X} \mathsf{N}(x, \mathcal{H}(x))\ *\ H = \bigodot_{x \in X} \mathcal{H}(x)$$

Gr is also implicitly parameterised by $fs$, edge and $\gamma$. The existentially-quantified $\mathcal{H}$ is a logical variable representing a *function* from nodes in $X$ to corresponding singleton flow graphs. $\mathsf{Gr}(X, H)$ describes a set of nodes $X$, such that each $x \in X$ is an $\mathsf{N}$ (in particular, it satisfies $\gamma$), whose singleton flow graphs compose back to $H$. As well as carrying ownership of the underlying heap locations, Gr's definition allows us to connect a node-level view of the region $X$ (each $\mathcal{H}(x)$) with a composite-level view defined by $H$, on which we can impose appropriate graph-level properties such as constraints on the region's inflow.

*Lifting to Interfaces.* Flow based proofs can often be expressed more elegantly and abstractly using predicates in terms of node and composite-level interfaces rather than flow graphs. To this end, we overload both our node and graph predicates with analogues whose second parameter is a flow interface, defined as follows:

$$\begin{aligned} \mathsf{N}(x, I) &:= \exists H.\ \mathsf{N}(x, H) * I = \mathsf{int}(H) \\ \mathsf{Gr}(X, I) &:= \exists H.\ \mathsf{Gr}(x, H) * I = \mathsf{int}(H) \end{aligned}$$

We will use these versions in the PIP proof below; interfaces capture all relevant properties for decomposition and composition of these flow graphs.

*Flow Lemmas.* We first illustrate our $\mathsf{N}$ and Gr predicates (which capture SL ownership of heap regions and abstract these with flow interfaces) by identifying a number of lemmas which are generically useful in flow-based proofs. Reasoning at the level of flow interfaces is entirely in the *pure* world (mathematics independent of heap-ownership and

[9] For simplicity, we assume that all fields of a flow graph node are to be handled by our flow-based technique, and that their ownership (via $\mapsto$ points-to predicates) is always carried around together; lifting these restrictions would be straightforward.

$$
\begin{array}{rcll}
\mathsf{Gr}(X_1 \uplus X_2, H) & \models & \exists H_1, H_2.\, \mathsf{Gr}(X_1, H_1) * \mathsf{Gr}(X_2, H_2) & \\
& & * \; H_1 \odot H_2 = H & \text{(DECOMP)} \\
\mathsf{Gr}(X_1, H_1) * \mathsf{Gr}(X_2, H_2) * H_1 \odot H_2 \neq \bot & \models & \mathsf{Gr}(X_1 \uplus X_2, H_1 \odot H_2) & \text{(COMP)} \\
\mathsf{N}(x, H) & \equiv & \mathsf{Gr}(\{x\}, H) & \text{(SING)} \\
emp & \models & \mathsf{Gr}(\emptyset, H_\emptyset) & \text{(GREMP)} \\
\mathsf{Gr}(X_1, H_1') * \mathsf{Gr}(X_2, H_2) * H = H_1 \odot H_2 & \models & \mathsf{Gr}(X_1 \uplus X_2, H_1' \odot H_2) & \text{(REPL)} \\
* \; \mathsf{int}(H_1) = \mathsf{int}(H_1') & & * \; \mathsf{int}(H) = \mathsf{int}(H_1' \odot H_2) &
\end{array}
$$

Fig. 2: Some useful lemmas for proving entailments between flow-based specifications.

resources) with respect to the underlying SL reasoning; these lemmas are consequences of our predicate definitions and the foundational flow framework definitions themselves.

Examples of these lemmas are shown in Figure 2. (DECOMP) shows that we can always decompose a valid flow graph into subgraphs which are themselves flow graphs. Recomposition (COMP) is possible only if the subgraphs compose. These rules, as well as (SING), and (GREMP) follow directly from the definition of Gr and standard SL properties of iterated separating conjunction. The final rule (REPL) is a direct consequence of rules (COMP), (DECOMP) and the congruence relation on flow graphs induced by their interfaces (cf. Lemma 2). Conceptually, it expresses that after decomposing any flow graph into two parts $H_1$ and $H_2$, we can *replace* $H_1$ with a new flow graph $H_1'$ with the same interface; when recomposing, the overall graph will be a flow graph with the same overall interface.

Note the connection between rules (COMP)/(DECOMP) and the algebraic laws of standard inductive predicates such as ls describing a segment of a linked list [2]. For instance by combining the definition of Gr with these rules and (SING) we can prove the following graph analogue of the rule to separate a list into the head node and the tail:

$$\mathsf{Gr}(X \uplus \{y\}, H) \equiv \exists H_y, H'.\mathsf{N}(y, H_y) * \mathsf{Gr}(X, H') * H = H_y \odot H' \quad \text{((UN)FOLD)}$$

However, crucially (and unlike when using general inductive predicates [32]), this rule is symmetrical for any node $x$ in $X$; it works analogously for any desired order of decomposition of the graph, and for any data structure specified using flows.

When working with our overloaded N and Gr predicates, similar steps to those described by the above lemmas are useful. Given these overloaded predicates, we simply apply the lemmas above to the *existentially quantified* flow-graphs in their definitions and then lift the consequence of the lemma back to the interface level using the congruence between our flow graph and interface composition notions (Lemma 2).

#### 3.2 Proof of the PIP

We now have all the tools necessary to verify the priority inheritance protocol (PIP). Figure 3 gives the full algorithm with flow-based specifications; we also include some intermediate assertions to illustrate the reasoning steps for the `acquire` method, which

```
1  // Let $\delta(m, q_1, q_2) \coloneqq m \setminus (q_1 \geq 0\ ?\ \{q_1\} : \emptyset) \cup (q_2 \geq 0\ ?\ \{q_2\} : \emptyset)$
2
3  method update(n: Ref, from: Int, to: Int)
4    requires $\mathsf{N}(n, I_n) * \mathsf{Gr}(X \setminus \{n\}, I') * I = I'_n \oplus I' * \varphi(I) * n \in X$
5    requires $I'_n = (\{n \rightarrowtail \delta(I_n.in(n), from, to)\}, I_n.out) * from \neq to$
6    ensures $\mathsf{Gr}(X, I)$
7  {
8    n.prios := n.prios \ {from}
9    if (to >= 0) {
10     n.prios := n.prios ∪ {to}
11   }
12   from := n.curr_prio
13   n.curr_prio := max(n.prios ∪ {n.def_prio})
14   to := n.curr_prio
15
16   if (from != to && n.next != null) {
17     update(n.next, from, to)
18   }
19 }
20
21 method acquire(p: Ref, r: Ref)
22   requires $\mathsf{Gr}(X, I) * \varphi(I) * p \in X * r \in X * p \neq r$
23   ensures $\mathsf{Gr}(X, I)$
24 {
25   $\{\exists I_r, I_p, I_1.\ \mathsf{N}(r, I_r) * \mathsf{N}(p, I_p) * \mathsf{Gr}(X \setminus \{r, p\}, I_1) * I = I_r \oplus I_p \oplus I_1 * \varphi(I)\}$
26   if (r.next == null) {
27     r.next := p;
28     // Let $q_r$ = r.curr_prio
29     $\left\{\begin{array}{l} \exists I_r, I'_r, I_p, I_1.\ \mathsf{N}(r, I'_r) * \mathsf{N}(p, I_p) * \mathsf{Gr}(X \setminus \{r, p\}, I_1) * I = I_r \oplus I_p \oplus I_1 \\ * I'_r = (I_r.in, \{p \rightarrowtail \{q_r\}\}) * I_r.out = \lambda_0 * \cdots \end{array}\right\}$
30     $\models \left\{\begin{array}{l} \exists I_p, I'_p, I_2.\ \mathsf{N}(p, I_p) * \mathsf{Gr}(X \setminus \{p\}, I_2) * I = I'_p \oplus I_2 \\ * I'_p = (\{p \rightarrowtail \delta(I_p.in(p), -1, q_r)\}, I_p.out) * \cdots \end{array}\right\}$
31     update(p, -1, r.curr_prio)
32     $\{\mathsf{Gr}(X, I)\}$
33   } else {
34     p.next := r; update(r, -1, p.curr_prio)
35   }
36 }
37
38 method release(p: Ref, r: Ref)
39   requires $\mathsf{Gr}(X, I) * \varphi(I) * p \in X * r \in X * p \neq r$
40   ensures $\mathsf{Gr}(X, I)$
41 { r.next := null; update(p, r.curr_prio, -1) }
```

Fig. 3: Full PIP code and specifications, with proof sketch for `acquire`. The comments and coloured annotations (lines 29 to 32) are used to highlight steps in the proof, and are explained in detail in the text.

we explain in more detail below. [10] We instantiate our framework in order to capture the PIP invariants as follows:

$$fs := \{\texttt{next}\colon y,\ \texttt{curr\_prio}\colon q,\ \texttt{def\_prio}\colon q^0,\ \texttt{prios}\colon Q\}$$

$$\mathsf{edge}(x, fs, z) := \begin{cases} (\lambda m.\ \max(m \cup \{q^0\})) & \text{if } z = y \neq \mathit{null} \\ \lambda_0 & \text{otherwise} \end{cases}$$

$$\gamma(x, fs, m) := q^0 \geq 0 \ * \ (\forall q' \in Q.\ q' \geq 0) \ * \ m = Q \ * \ q = \{\max(Q \cup \{q^0\})\}$$

$$\varphi(I) := I = (\lambda_0, \lambda_0)$$

Each node has the four fields listed in *fs*. *fs* also defines variables such as $y$ to denote field values that are used in the definitions of edge and $\gamma$; these variables are bound to the heap by N. edge abstracts the heap into a flow graph by letting each node have an edge to its `next` successor labelled by a function that passes to it the maximum incoming priority or the node's default priority: whichever is larger. With this definition, one can see that the flow of every node will be the multiset containing exactly the priorities of its predecessors. The node-local invariant $\gamma$ says that all priorities are non-negative, the flow $m$ of each node is stored in the `prios` field, and its current priority is the maximum of its default and incoming priorities. Finally, the constraint $\varphi$ on the global interface expresses that the graph is closed – it has no inflow or outflow.

*Flows Specifications for the PIP.* Our specifications of `acquire` and `release` guarantee that if we start with a valid flow graph (closed, according to $\varphi$), we are guaranteed to return a valid flow graph with the same interface (i.e. the graph remains closed). For clarity of the exposition, we focus here on how we prove that being a flow graph that satisfies the PIP invariant is preserved (as is the composite flow graph's interface). Extending this specification to one which proves, e.g., that `acquire` adds the expected edge is straightforward (see Appendix C of the TR [23]). [11]

The specification for `update` is somewhat subtle, and exploits the full flexibility of flow interfaces as a specification medium. The preconditions of `update` describe an update to the graph which is not yet completed. There are three complementary aspects to this specification. Firstly, (as for `acquire` and `release`), node-local invariants ($\gamma$) hold for all nodes in the graph (enforced via N and Gr predicates). Secondly, we employ flow interfaces to express a decomposition of the original top-level interface $I$ into compatible (primed) sub-interfaces. The key to understanding this specification is that $I'_n$ is in some sense a *fake* interface; it does not abstract the current state of the heap node $n$. Instead, $I'_n$ expresses the way in which the node $n$'s current inflow *hasn't yet* been accounted for in the heap: that *if* $n$ could adjust its inflow according to the propagated priority change *without* changing its outflow, then it would compose back with the rest of the graph, and restore the graph's overall interface. The shorthand $\delta$ defines the required change to $n$'s inflow.

In general (except when $n$'s `next` field is null, or $n$'s flow value is unchanged), it is not even possible for $n$'s fields to be updated to satisfy $I'_n$; by updating $n$'s inflow,

[10] In specifications, we implicitly quantify at the top level over free variables such as $I$. $\lambda_0$ denotes an identically zero function on an unconstrained domain.

[11] We also omit `acquire`'s precondition that `p.next == null` for brevity.

we will necessarily update its outflow. However, we can then construct a corresponding "fake" interface for the next node in the graph, reflecting the update yet to be accounted for, and establishing the precondition for the recursive call to `update`.

The third specification aspect is the *connection* between heap-level nodes and interfaces. The $\mathsf{N}(n, I_n)$ predicate connects $n$ with a *different* interface; $I_n$ is the actual current abstraction of $n$'s state. Conceptually, the key property which is broken at this point is this connection between the interface-level specification and the heap at node $n$, reflected by the decomposition in the specification between $X \setminus \{n\}$ and $\{n\}$.

We note that the same specification ideas and proof style can be easily adapted to other data structure implementations with an update-notify style, including well-known designs such as Subject-Observer patterns, or the Composite pattern [27].

*Proof Outline.* To illustrate the application of flows reasoning to our PIP specification ideas more clearly, we examine in detail the first `if`-branch in the proof of `acquire`. Our intermediate proof steps are shown as purple annotations surrounded by braces. The first step, as shown in the first line inside the method body, is to apply ((UN)FOLD) twice (on the flow graphs represented by these predicates) and peel off $\mathsf{N}$ predicates for each of $r$ and $p$. The update to $r$'s `next` field (line 27) causes the correct singleton interface of $r$ to change to $I'_r$: its outflow (previously none, since the `next` field was null) now propagates flow to $p$. We summarise this state in the assertion on line 29 (we omit e.g. repetition of properties from the function's precondition, focusing on the flow-related steps of the argument). We now rewrite this state; using the definition of interface composition (Definition 6) we deduce that although $I'_r$ and $I_p$ do not compose (since the former has outflow that the latter does not account for as inflow), the alternative "fake" interface $I'_p$ for $p$ (which artificially accounts for the missing inflow) *would* do so (cf. line 30). Essentially, we show $I_r \oplus I_p = I'_r \oplus I'_p$, that the interface of $\{r, p\}$ would be unchanged if $p$ could somehow have interface $I'_p$. Now by setting $I_2 = I'_r \oplus I_1$ and using algebraic properties of interfaces, we assemble the precondition expected by `update`. After the call, `update`'s postcondition gives us the desired postcondition.

We focused here on the details of `acquire`'s proof, but very similar manipulations are required for reasoning about the recursive call in `update`'s implementation.[12] The main difference there is that if the if-condition wrapping the recursive call is false then either the last-modified node has no successor (and so there is no outstanding inflow change needed), or we have `from` $=$ `to` which implies that the "fake" interface is actually the same as the currently correct one.

Despite the property proved for the PIP example being a rather delicate recursive invariant over the (potentially cyclic) graph, the power of our framework enables extremely succinct specifications for the example, and proofs which require the application of relatively few generic lemmas. The integration with standard separation logic reasoning, and the complementary separation algebras provided by flow interfaces allow decomposition and recomposition to be simple proof steps. For this proof, we integrated with standard sequential separation logic, but in the next section we will show that compatibility with concurrent SL techniques is similarly straightforward.

[12] We provide further proof outlines in Appendix C of the TR [23].

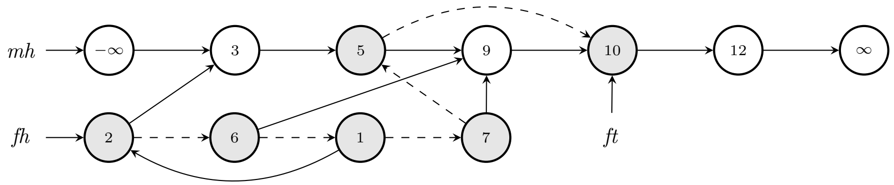

Fig. 4: A potential state of the Harris list with explicit memory management. `fnext` pointers are shown with dashed edges, marked nodes are shaded gray, and null pointers are omitted for clarity.

### 4 Advanced Flow Reasoning and the Harris List

This section introduces some advanced foundational flow framework theory and demonstrates its use in the proof of the Harris list. We note that [22] presented a proof of this data structure in the original flow framework. The proof given here shows that the new framework eliminates the need for the customized concurrent separation logic defined in [22]. We start with a recap of Harris' algorithm adapted from [22].

#### 4.1 The Harris List Algorithm

The power of flow-based reasoning is exhibited in the proof of overlaid data structures such as the Harris list, a concurrent non-blocking linked list algorithm [12]. This algorithm implements a set data structure as a sorted list, and uses atomic compare-and-swap (CAS) operations to allow a high degree of parallelism. As with the sequential linked list, Harris' algorithm inserts a new key $k$ into the list by finding nodes $k_1, k_2$ such that $k_1 < k < k_2$, setting $k$ to point to $k_2$, and using a CAS to change $k_1$ to point to $k$ only if it was still pointing to $k_2$. However, a similar approach fails for the delete operation. If we had consecutive nodes $k_1, k_2, k_3$ and we wanted to delete $k_2$ from the list (say by setting $k_1$ to point to $k_3$), there is no way to ensure with one CAS that $k_2$ and $k_3$ are also still adjacent (another thread could have inserted/deleted in between them).

Harris' solution is a two step deletion: first atomically mark $k_2$ as deleted (by setting a mark bit on its successor field) and then later remove it from the list using a single CAS. After a node is marked, no thread can insert or delete to its right, hence a thread that wanted to insert $k'$ to the right of $k_2$ would first remove $k_2$ from the list and then insert $k'$ as the successor of $k_1$.

In a non-garbage-collected environment, unlinked nodes cannot be immediately freed as suspended threads might continue to hold a reference to them. A common solution is to maintain a second "free list" to which marked nodes are added before they are unlinked from the main list (this is the so-called drain technique). These nodes are then labelled with a timestamp, which is used by a maintenance thread to free them when it is safe to do so. This leads to the kind of data structure shown in Figure 4, where each node has two pointer fields: a `next` field for the main list and an `fnext` field for the free list (the list from *fh* to *ft* via dashed edges). Threads that have been suspended while holding

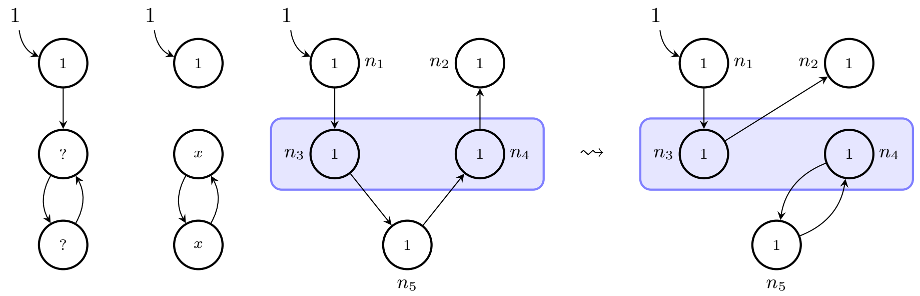

Fig. 5: Examples of graphs that motivate effective acyclicity. All graphs use the path-counting flow domain, the flow is displayed inside each node, and the inflow is displayed as curved arrows to the top-left of nodes. (a) shows a graph and inflow that has no solution to (FlowEqn); (b) has many solutions. (c) shows a modification that preserves the interface of the modified nodes, yet goes from a graph that has a unique flow to one that has many solutions to (FlowEqn).

a reference to a node that was added to the free list can simply continue traversing the `next` pointers to find their way back to the unmarked nodes of the main list.

Even for seemingly simple properties such as that the Harris list is memory safe and not leaking memory, the proof will rely on the following non-trivial invariants:

(a) The data structure consists of two (potentially overlapping) lists: a list on `next` edges beginning at $mh$ and one on `fnext` edges beginning at *fh*.
(b) The two lists are null terminated and `next` edges from nodes in the free list point to nodes in the free list or main list.
(c) All nodes in the free list are marked.
(d) *ft* is an element in the free list (due to concurrency, it's not always the tail).

*Challenges.* To prove that Harris' algorithm maintains the invariants listed above we must tackle a number of challenges. First, we must construct flow domains that allow us to describe overlaid data structures, such as the overlapping main and free lists (§4.2). Second, the flow-based proofs we have seen so far work by showing that the interface of some modified region is unchanged. However, if we consider a program that allocates and inserts a new node into a data structure (like the insert method of Harris), then the interface cannot be the same since the domain has changed (it has increased by the newly allocated node). We must thus have a means to reason about preservation of flows by modifications that allocate new nodes (§4.3). The third issue is that in some flow domains, there exist graphs $G$ and inflows $in$ for which no solutions to the flow equation (FlowEqn) exist. For instance, consider the path-counting flow domain and the graph in Figure 5(a). Since we would need to use the path-counting flow in the proof of the Harris list to encode its structural invariants, this presents a challenge (§4.4).

We will next see how to overcome these three challenges in turn, and then apply those solution to the proof of the Harris list in §4.5.

#### 4.2 Product Flows for Reasoning about Overlays

An important fact about flows is that any flow of a graph over a product of two flow domains is the product of the flows on each flow domain component.

**Lemma 3.** *Given two flow domains* $(M_1, +_1, 0_1, E_1)$ *and* $(M_2, +_2, 0_2, E_2)$*, the* product *domain* $(M_1 \times M_2, +, (0_1, 0_2), E)$ *is a flow domain, where* $+$ *and* $E$ *are the pointwise liftings of* $(+_1, +_2)$ *and* $(E_1, E_2)$*, respectively, to the domain* $M_1 \times M_2$*.*

This lemma greatly simplifies reasoning about overlaid graph structures; we will use the product of two path-counting flows to describe a structure consisting of two overlaid lists that make up the Harris list.

#### 4.3 Contextual Extensions and the Replacement Theorem

In general, when modifying a flow graph $H$ to another flow graph $H'$, requiring that $H'$ satisfies *precisely* the same interface $\mathsf{int}(H)$ can be too strong a condition as it does not permit allocating new nodes. Instead, we want to allow $\mathsf{int}(H')$ to differ from $\mathsf{int}(H)$ in that the new interface could have a larger domain, as long as the edges from the new nodes do not change the outflow of the modified region.

**Definition 7.** *An interface* $I = (in, out)$ *is* contextually extended *by* $I' = (in', out')$*, written* $I \precsim I'$*, if and only if the following conditions all hold:*

*(1)* $\mathsf{dom}(in) \subseteq \mathsf{dom}(in')$*,*
*(2)* $\forall n \in \mathsf{dom}(in).\ in(n) = in'(n)$*, and*
*(3)* $\forall n' \notin \mathsf{dom}(in').\ out(n') = out'(n')$*.*

The following theorem states that contextual extension preserves composability and is itself preserved under interface composition.

**Theorem 2 (Replacement Theorem).** *If* $I = I_1 \oplus I_2$*, and* $I_1 \precsim I_1'$ *are all valid interfaces such that* $I_1' \cap I_2 = \emptyset$ *and* $\forall n \in I_1' \setminus I_1.\ I_2.out(n) = 0$*, then there exists a valid* $I' = I_1' \oplus I_2$ *such that* $I \precsim I'$*.*

In terms of our flow predicates, this theorem gives rise to the following adaptation of the (REPL) rule:

$$\begin{aligned} &\mathsf{Gr}(X_1', H_1') * \mathsf{Gr}(X_2, H_2) * H = H_1 \odot H_2 * \mathsf{int}(H_1) \precsim \mathsf{int}(H_1') \\ \models\ &\exists H'.\ \mathsf{Gr}(X_1' \uplus X_2, H') * H' = H_1' \odot H_2 * \mathsf{int}(H) \precsim \mathsf{int}(H') \qquad \text{(REPL+)} \end{aligned}$$

The rule (REPL+) is derived from the Replacement Theorem by instantiating with $I = \mathsf{int}(H), I_1 = \mathsf{int}(H_1), I_2 = \mathsf{int}(H_2)$ and $I_1' = \mathsf{int}(H_1')$. We know $I_1 \precsim I_1'$; $H = H_1 \odot H_2$ tells us (by Lemma 2) that $I = I_1 \oplus I_2$, and $\mathsf{Gr}(X_1', H_1') * \mathsf{Gr}(X_2, H_2)$ gives us $I_1' \cap I_2 = \emptyset$. The final condition of the Replacement Theorem is to prove that there is no outflow from $X_2$ to any newly allocated node in $X_1'$. While we can use additional ghost state to prove such constraints in our proofs, if we assume that the memory allocator only allocates fresh addresses and restrict the abstraction function edge to only propagate flow along an edge $(n, n')$ if $n$ has a (non-ghost) field with a reference to $n'$ then this condition is always true. For simplicity, and to keep the focus of this paper on the flow reasoning, we make this assumption in the Harris list proof.

#### 4.4 Existence and Uniqueness of Flows

We typically express global properties of a graph $G = (N, e)$ by fixing a global inflow $in\colon N \to M$ and then constraining the flow of each node in $N$ using node-local conditions. However, as we discussed at the beginning of this section, there is no general guarantee that a flow exists or is unique for a given $in$ and $G$. The remainder of this section presents two complementary conditions under which we can prove that our flow fixpoint equation always has a unique solution. To this end, we say that a flow domain $(M, +, 0, E)$ has *unique flows* if for every graph $(N, e)$ over this flow domain and inflow $in\colon N \to M$, there exists a unique $fl$ that satisfies the flow equation $\mathsf{FlowEqn}(in, e, fl)$. But first, we briefly recall some more monoid theory.

We say $M$ is *positive* if $m_1 + m_2 = 0$ implies that $m_1 = m_2 = 0$. For a positive monoid $M$, we can define a partial order $\leq$ on its elements as $m_1 \leq m_2$ if and only if $\exists m_3.\ m_1 + m_3 = m_2$. This definition implies that every $m \in M$ satisfies $0 \leq m$.

For $e, e'\colon M \to M$, we write $e + e'$ for the function that maps $m \in M$ to $e(m) + e'(m)$. We lift this construction to a set of functions $E$ and write it as $\sum_{e \in E} e$.

**Definition 8.** *A function $e\colon M \to M$ is called an* endomorphism *on $M$ if for every $m_1, m_2 \in M$, $e(m_1 + m_2) = e(m_1) + e(m_2)$. We denote the set of all endomorphisms on $M$ by $\mathsf{End}(M)$.*

Note that for cancellative $M$, $e(0) = 0$ for every endomorphism $e \in \mathsf{End}(M)$. Note further that $e + e' \in \mathsf{End}(M)$ for any $e, e' \in \mathsf{End}(M)$. Similarly, for finite sets $E \subseteq \mathsf{End}(M)$, $\sum_{e \in E} e \in \mathsf{End}(M)$. We say that a set of endomorphisms $E \subseteq \mathsf{End}(M)$ is *closed* if for every $e, e' \in E$, $e \circ e' \in E$ and $e + e' \in E$.

*Nilpotent Cycles.* Let $(M, +, 0, E)$ be a flow domain where every edge function $e \in E$ is an endomorphism on $M$. In this case, we can show that the flow of a node $n$ is the sum of the flow as computed along *each path* in the graph that ends at $n$. Suppose we additionally know that the edge functions are defined such that their composition along any *cycle* in the graph eventually becomes the identically zero function. We then need only consider finitely many paths to compute the flow of a node, which means the flow equation has a unique solution.

**Definition 9.** *A closed set of endomorphisms $E \subseteq \mathsf{End}(M)$ is called* nilpotent *if there exists $p > 1$ such that $e^p \equiv 0$ for every $e \in E$.*

*Example 5.* The flow domain $(\mathbb{N}^2, +, (0,0), \{(\lambda(x,y).\ (0, c \cdot x)) \mid c \in \mathbb{N}\})$ contains nilpotent edge functions that shift the first component of the flow to the second (with a scaling factor). This domain can be used to express the property that every node in a graph is reachable from the root via a single edge (by requiring the flow of every node to be $(0,1)$ under the inflow $(\lambda n.\ (n = r\ ?\ (1,0) : (0,0)))$).

Before we prove that nilpotent endomorphisms lead to unique flows, we present a useful notion when dealing with endomorphic flow domains.

**Definition 10.** *The* capacity *of a flow graph $G = (N, e)$ is $\mathsf{cap}(G)\colon N \times \mathfrak{N} \to (M \to M)$, defined inductively as $\mathsf{cap}(G) := \mathsf{cap}^{|G|}(G)$, where $\mathsf{cap}^0(G)(n, n') := \delta_{n=n'}$ and*

$$\mathsf{cap}^{i+1}(G)(n, n') := \delta_{n=n'} + \sum_{n'' \in G} \mathsf{cap}^i(G)(n, n'') \circ e(n'', n').$$

For a flow graph $H = (N, e, \mathit{fl})$, we write $\mathsf{cap}(H)(n, n') = \mathsf{cap}((N, e))(n, n')$ for the capacity of the underlying graph. Intuitively, $\mathsf{cap}(G)(n, n')$ is the function that summarizes how flow is routed from any source node $n$ in $G$ to any other node $n'$, including those outside of $G$.

We can now show that if all edges of a flow graph are labelled with edges from a nilpotent set of endomorphisms, then the flow equation has a unique solution:

**Lemma 4.** *If $(M, +, 0, E)$ is a flow domain such that $M$ is a positive monoid and $E$ is a nilpotent set of endomorphisms, then this flow domain has unique flows.*

*Effectively Acyclic Flow Graphs.* There are some flow domains that compute flows useful in practice, but which do not guarantee either existence or uniqueness of fixpoints *a priori* for all graphs. For example, the path-counting flow from Example 1 is one where for certain graphs, there exist no solutions to the flow equation (see Figure 5(a)), and for others, there can exist more than one (in Figure 5(b), the nodes marked with $x$ can have any path count, as long as they both have the same value).

In such cases, we explore how to restrict the class of *graphs* we use in our flow-based proofs such that each graph has a unique fixpoint; the difficulty is that this restriction must be respected for composition of our graphs. Here, we study the class of flow domains $(M, +, 0, E)$ such that $M$ is a positive monoid and $E$ is a set of *reduced* endomorphisms (defined below). In such domains we can decompose the flow computations into the various paths in the graph, and achieve unique fixpoints by restricting the kinds of cycles graphs can have.

**Definition 11.** *A flow graph $H = (N, e, \mathit{fl})$ is* effectively acyclic (EA) *if for every $1 \leq k$ and $n_1, \ldots, n_k \in N$,*

$$\mathit{fl}(n_1) \triangleright e(n_1, n_2) \cdots e(n_{k-1}, n_k) \triangleright e(n_k, n_1) = 0.$$

The simplest example of an effectively acyclic graph is one where the edges with non-zero edge functions form an acyclic graph. However, our semantic condition is weaker: for example, when reasoning about two overlaid acyclic lists whose union happens to form a cycle, a product of two path-counting domains will satisfy effective acyclicity because the composition of different types of edges results in the zero function.

**Lemma 5.** *Let $(M, +, 0, E)$ be a flow domain such that $M$ is a positive monoid and $E$ is a closed set of endomorphisms. Given a graph $(N, e)$ over this flow domain and inflow $in \colon N \to M$, if there exists a flow graph $H = (N, e, \mathit{fl})$ that is effectively acyclic, then $\mathit{fl}$ is unique.*

While the restriction to effectively acyclic flow graphs guarantees us that the flow is the unique fixpoint of the flow equation, it is not easy to show that modifications to the graph preserve EA while reasoning locally. Even modifying a subgraph to another with the same flow interface (which we know guarantees that it will compose with any context) can inadvertently create a cycle in the larger composite graph. For instance, consider Figure 5(c), that shows a modification to nodes $\{n_3, n_4\}$ (the boxed blue region). The interface of this region is $(\{n_3 \rightarrowtail 1, n_4 \rightarrowtail 1\}, \{n_5 \rightarrowtail 1, n_2 \rightarrowtail 1\})$, and so swapping the edges of $n_3$ and $n_4$ preserves this interface. However, the resulting graph, despite composing with the context to form a valid flow graph, is not EA (in this case, it has multiple solutions to the flow equation). This shows that flow interfaces are not powerful enough to preserve effective acyclicity. For a special class of endomorphisms, we show that a local property of the modified subgraph can be checked, which implies that the modified composite graph continues to be EA.

**Definition 12.** *A closed set of endomorphisms $E \subseteq \mathsf{End}(M)$ is called* reduced *if $e \circ e \equiv \lambda_0$ implies $e \equiv \lambda_0$ for every $e \in E$.*

Note that if $E$ is reduced, then no $e \in E$ can be nilpotent. In that sense, this class of instantiations is complementary to the nilpotent class.

*Example 6.* Examples of flow domains that fall into this class include positive semirings of reduced rings (with the additive monoid of the semiring being the aggregation monoid of the flow domain and $E$ being any set of functions that multiply their argument with a constant flow value). Note that any direct product of integral rings is a reduced ring. Hence, products of the path counting flow domain are a special case.

For reduced endomorphisms, it suffices to check that a modification preserves the flow routed between every pair of source and sink node in order to ensure that it does not create any new cycles in any composite graph.

**Definition 13.** *A flow graph $H'$ is a* subflow-preserving extension *of $H$, for which we write $H \precsim_s H'$, if the following conditions all hold:*

*(1)* $\mathsf{int}(H) \precsim \mathsf{int}(H')$
*(2)* $\forall n \in H, n' \notin H', m.\ m \leq \mathsf{inf}(H)(n) \Rightarrow m \triangleright \mathsf{cap}(H)(n, n') = m \triangleright \mathsf{cap}(H')(n, n')$
*(3)* $\forall n \in H' \setminus H, n' \notin H', m.\ m \leq \mathsf{inf}(H')(n) \Rightarrow m \triangleright \mathsf{cap}(H')(n, n') = 0$

This pairwise check, apart from requiring the interface of the modified region to be unchanged, also permits allocating new nodes as long as no flow is routed via the new nodes (condition (3)). We now show that it is sufficient to check that a modification is a subflow-preserving extension to guarantee composition back to an effectively-acyclic composite graph:

**Theorem 3.** *Let $(M, +, 0, E)$ be a flow domain such that $M$ is a positive monoid and $E$ is a reduced set of endomorphisms. If $H = H_1 \odot H_2$ and $H_1 \precsim_s H_1'$ are all effectively acyclic flow graphs such that $H_1' \cap H_2 = \emptyset$ and $\forall n \in H_1' \setminus H_1.\ \mathsf{outf}(H_2)(n) = 0$, then there exists an effectively acyclic flow graph $H' = H_1' \odot H_2$ such that $H \precsim_s H'$.*

We define effectively acyclic versions of our flow graph predicates, $\mathsf{N_a}(x, H)$ and $\mathsf{Gr_a}(X, H)$, that additionally constrain $H$ to be effectively acyclic. The above theorem yields the following variant of the (REPL) rule for EA graphs:

$$\begin{array}{rl} & \mathsf{Gr_a}(X_1', H_1') * \mathsf{Gr_a}(X_2, H_2) * H = H_1 \odot H_2 * H_1 \precsim_s H_1' \\ \models & \exists H'.\ \mathsf{Gr_a}(X_1' \uplus X_2, H') * H' = H_1' \odot H_2 * H \precsim_s H' \end{array} \quad \text{(REPLEA)}$$

#### 4.5 Proof of the Harris List

We use the techniques seen in this section in the proof of the Harris list. As the data structure consists of two potentially overlapping lists, we use Lemma 3 to construct a product flow domain of two path-counting flows: one tracks the path count from the head of the main list, and one from the head of the free list. We also work under the effectively acyclic restriction (i.e. we use the $\mathsf{N_a}$ and $\mathsf{Gr_a}$ predicates), both in order to obtain the desired interpretation of the flow as well as to ensure existence of flows in this flow domain.

We instantiate the framework using the following definitions of parameters:

$$
\begin{aligned}
fs &:= \{\mathtt{key}\colon k, \mathtt{next}\colon y, \mathtt{fnext}\colon z\}\\
\mathsf{edge}(x, fs, v) &:= (v = null\ ?\ \lambda_0 : (v = y \wedge y \neq z\ ?\ \lambda_{(1,0)}\\
&\qquad : (v \neq y \wedge y = z\ ?\ \lambda_{(0,1)} : (v = y \wedge y = z\ ?\ \lambda_{\mathsf{id}} : \lambda_0))))\\
\gamma(x, fs, I) &:= (I.in(x) \in \{(1,0),(0,1),(1,1)\}) * (I.in(x) \neq (1,0) \Rightarrow M(y))\\
&\qquad * (x = ft \Rightarrow I.in(x) = (\_, 1)) * (\neg M(y) \Rightarrow z = null)\\
\varphi(I) &:= I = (\lambda_0[mh \rightarrowtail (1,0)][fh \rightarrowtail (0,1)], \lambda_0)
\end{aligned}
$$

Here, edge encodes the edge functions needed to compute the product of two path counting flows, the first component tracks path-counts from $mh$ on `next` edges and the second tracks path-counts from $fh$ on `fnext` edges [13]. The node-local invariant $\gamma$ says: the flow is one of $\{(1,0),(0,1),(1,1)\}$ (meaning that the node is on one of the two lists, invariant (a)); if the flow is not $(1,0)$ (the node is not only on the main list, i.e. it is on the free list) then the node is marked (indicated by $M(y)$, invariant (c)); and if the node is $ft$ then it must be on the free list (invariant (d)). The constraint on the global interface, $\varphi$, says that the inflow picks out $mh$ and $fh$ as the roots of the lists, and there is no outgoing flow (thus, all non-null edges must stay within the graph, invariant (b)).

Since the Harris list is a concurrent algortihm, we perform the proof in rely-guarantee separation logic (RGSep) [41]. Like in §3, we do not need to modify the semantics of RGSep in any way; our flow-based predicates can be defined and reasoning using our lemmas can be performed in the logic out-of-the-box. For space reasons, the full proof can be found in Appendix D of the TR [23].

### 5 Related Work

As mentioned in §1, the most closely related work is the flow framework developed by some of the authors in [22]. We here present a simplified and generalized meta theory of flows that makes the approach much more broadly applicable. There were a number of limitations of the prior framework that prevented its application to more general classes of examples.

First, [22] required flow domains to form a semiring; the analogue of edge functions are restricted to multiplication with a constant which must come from the same flow

[13] We use the shorthands $\lambda_{(1,0)} := (\lambda(m_1, m_2).\ (m_1, 0))$ and $\lambda_{(0,1)} := (\lambda(m_1, m_2).\ (0, m_2))$, and denote an anonymous existentially-quantified variable by _.

value set. This restriction made it complex to encode many graph properties of interest. For example, one could not easily encode the PIP flow, or a simple flow that counts the number of incoming edges to each node. Our foundational flow framework decouples the algebraic structure defining how flow is *aggregated* from the algebraic structure of the edge functions. In this way, we obtain a more general framework that applies to many more examples, and with simpler flow domains.

Second, in [22], a flow graph did not uniquely determine its inflow (cf. Lemma 1). Correspondingly, [22]'s notion of interface included an *equivalence class* of inflows (all those that induce the same flow values). Since, in [22], the interface also determines which modifications are permitted by the framework, [22] could only handle modifications that preserve the inflow equivalence class. For example, this prevents one from reasoning locally about the removal of a single edge from a graph in certain cases (in particular, like `release` does in the PIP). Our foundational flow framework solves this problem by requiring that the aggregation operation on flow values is cancellative, guaranteeing unique inflows.

Cancellativity is fundamentally incompatible with [22], which requires the flow domain to form an $\omega$-CPO in order to guarantee the existence of unique flows. For example, in a graph with two nodes $n$ and $n'$ with identity edges between them and all other edges zero (in [22], edges labelled with 1 and 0), if we have $in(n) = 0$ and $in(n) = m$ for some non-zero $m$, a solution to the flow equation must satisfy $fl(n) = m + fl(n)$. [22] forces such solutions to exist, ruling out cancellativity. To solve this problem, we present a new theory which can optionally guarantee unique flows when desired and show that requiring cancellativity does not limit expressivity.

Next, the proofs of programs shown in [22] depend on a bespoke program logic. This logic requires new reasoning primitives that are not supported by the logics implemented in existing SL-based verification tools. Our general proof technique eliminates the need for a dedicated program logic and can be implemented on top of standard separation logics and existing SL-based tools. Finally, the underlying separation algebra of the original framework makes it hard to use equational reasoning, which is a critical prerequisite for enabling proof automation.

An abundance of SL variants provide complementary mechanisms for modular reasoning about programs (e.g. [18,36,38]). Most are parameterized by the underlying separation algebra; our flow-based reasoning technique easily integrates with these existing logics.

The most common approach to reason about irregular graph structures in SL is to use iterated separating conjunction [30,44] and describe the graph as a set of nodes each of which satisfies some local invariant. This approach has the advantage of being able to naturally describe general graphs. However, it is hard to express non-local properties that involve some form of fixpoint computation over the graph structure. One approach is to abstract the program state as a mathematical graph using iterated separating conjunction and then express non-local invariants in terms of the abstract graph rather than the underlying program state [14,35,38]. However, a proof that a modification to the state maintains a global invariant of the abstract graph must then often revert back to non-local and manual reasoning, involving complex inductive arguments about paths, transitive closure, and so on. Our technique also exploits iterated separating conjunction for the underlying heap ownership, with the key benefit that flow interfaces exactly capture the necessary conditions on a modified subgraph in order to compose with *any* context and preserve desired non-local invariants.

In recent work, Wang et al. present a Coq-mechanised proof of graph algorithms in C, based on a substantial library of graph-related lemmas, both for mathematical and heap-based graphs [42]. They prove rich functional properties, integrated with the VST tool. In contrast to our work, a substantial suite of lemmas and background properties are necessary, since these specialise to particular properties such as reachability. We believe that our foundational flow framework could be used to simplify framing lemmas in a way which remains parameteric with the property in question.

Proofs of a number of graph algorithms have been mechanized in various verification tools and proof assistants, including Tarjan's SCC algorithm [8], union-find [7], Kruskal's minimum spanning tree algorithm [13], and network flow algorithms [25]. These proofs generally involve non-local reasoning arguments about mathematical graphs.

An alternative approach to using SL-style reasoning is to commit to global reasoning but remain within decidable logics to enable automation [16,21,24,28,43]. However, such logics are restricted to certain classes of graphs and certain types of properties. For instance, reasoning about reachability in unbounded graphs with two successors per node is undecidable [15]. Recent work by Ter-Gabrielyan et al. [40] shows how to deal with modular framing of *pairwise reachability* specifications in an imperative setting. Their framing notion has parallels to our notion of interface composition, but allows subgraphs to *change* the paths visible to their context. The work is specific to a reachability relation, and cannot express the rich variety of custom graph properties available in our technique.

Dynamic frames [19] (e.g. implemented in Dafny [26]), can be used to explicitly reason about framing of heap information in a first-order logic. However, by itself, this theory does not enable modular reasoning about global graph properties. We believe that the flow framework could in principle be adapted to the dynamic frames setting.

### 6 Conclusions and Future Work

We have presented the foundational flow framework, enabling local modular reasoning about recursively-defined properties over general graphs. The core reasoning technique has been designed to make minimal mathematical requirements, providing great flexibility in terms of potential instantiations and applications. We identified key classes of these instantiations for which we can provide existence and uniqueness guarantees for the fixpoint properties our technique addresses and demonstrate our proof technique on several challenging examples. As future work, we plan to automate flow-based proofs in our new framework using existing tools that support SL-style reasoning such as Viper [29] and GRASShopper [34].

*Acknowledgments.* This work is funded in parts by the National Science Foundation under grants CCF-1618059 and CCF-1815633.
